# Inhibition of Bruton’s tyrosine kinase as a therapeutic strategy for chemoresistant oral squamous cell carcinoma and potential suppression of cancer stemness

**DOI:** 10.1038/s41389-021-00308-z

**Published:** 2021-02-27

**Authors:** Shao-Cheng Liu, Yang-Che Wu, Chih-Ming Huang, Ming-Shou Hsieh, Ting-Yi Huang, Chin-Sheng Huang, Tung-Nien Hsu, Mao-Suan Huang, Wei-Hwa Lee, Chi-Tai Yeh, Chun-Shu Lin

**Affiliations:** 1 Department of Otolaryngology-Head and Neck Surgery, Tri-Service General Hospital, National Defense Medical Center, Taipei City, 114 Taiwan; 2 School of Dentistry, College of Oral Medicine, Taipei Medical University, Taipei City, 110 Taiwan; 3 Department of Dentistry, Taipei Medical University—Shuang Ho Hospital, New Taipei City, 235 Taiwan; 4 Department of Otolaryngology, Taitung Mackay Memorial Hospital, Taipei City, Taiwan; 5 Department of Hematology and Oncology, Cancer Center, Taipei Medical University—Shuang Ho Hospital, New Taipei City, 235 Taiwan; 6 Department of Medical Research & Education, Taipei Medical University—Shuang Ho Hospital, New Taipei City, 235 Taiwan; 7 Department of Pathology, Taipei Medical University—Shuang Ho Hospital, New Taipei City, 235 Taiwan; 8 Department of Medical Laboratory Science and Biotechnology, Yuanpei University of Medical Technology, Hsinchu City, 30015 Taiwan; 9 Department of Radiation Oncology, Tri-Service General Hospital, National Defense Medical Center, Taipei City, 114 Taiwan

**Keywords:** Cancer stem cells, Head and neck cancer

## Abstract

Locally advanced oral squamous cell carcinoma (OSCC) requires multimodal therapy, including surgery and concurrent chemoradiotherapy (CCRT). CCRT-resistant and recurrent cancer has a poor prognosis. We investigated the effects of Bruton’s tyrosine kinase (BTK) on CCRT-resistant OSCC tissues. The effect of ibrutinib, a first-in-class BTK inhibitor, was tested on stem cell-like OSCC tumorspheres. A tissue array was constructed using tissue samples from 70 patients with OSCC. Human OSCC cell lines, SAS, TW2.6 and HSC-3, were examined. Wound healing, Matrigel invasion, and tumorsphere formation assays, as well as immunofluorescence analysis and flow cytometry, were used to investigate the effects of BTK knockdown (shBTK), ibrutinib, cisplatin, and ibrutinib/cisplatin combination on OSCC cells. We demonstrated that BTK was aberrantly highly expressed in the clinical CCRT-resistant OSCC tissue array, which resulted in poor overall survival in our local Tri-Service General Hospital and freely accessible TCGA OSCC cohorts. shBTK significantly downregulated the stemness markers Nanog, CD133, T cell immunoglobulin-3 (TIM-3), and Krüppel-like factor 4 (KLF4) in SAS tumorspheres and attenuated OSCC cell migration and colony formation. Ibrutinib reduced the number of aldehyde dehydrogenase (ALDH)-rich OSCC cells and reduced tumorsphere formation, migration, and invasion in a dose-dependent manner. Compared with ibrutinib or cisplatin monotherapy, the ibrutinib/cisplatin combination significantly reduced the formation of ALDH + OSCC tumorspheres and enhanced apoptosis. These results demonstrate that ibrutinib effectively inhibits the CSCs-like phenotype of OSCC cells through dysregulation of BTK/CD133 signaling. The ibrutinib/cisplatin combination may be considered for future clinical use.

## Introduction

Oral cancer, the sixth most diagnosed malignancy globally, is one of the most prevalent malignancies worldwide. Although the understanding of the pathogenesis, prevention, and treatment of head and neck cancers has increased over the last decade, no significant improvement has been observed in the survival rates of patients with oral carcinoma, and oral cancer still constitutes a major cause of cancer-associated mortality worldwide^1^. Oral squamous cell carcinoma (OSCC), accounting for >90% of all oral cancer cases, is a leading cause of disease-specific mortality, as recently reported in the 8th Edition of the American Joint Committee on Cancer staging system^2^. OSCC is particularly aggressive, as evidenced by considerable cell motility, invasiveness, and propensity for metastasis. Despite advances in the multimodal and interdisciplinary treatment of OSCC, patients’ prognosis remains unsatisfactory and particularly dismal in those with locally advanced, therapy-resistant, and recurrent disease. This necessitates improved insights into the causal biomolecular mechanism underlying aggressive therapy-resistant OSCC as well as the identification of novel druggable molecular drivers of OSCC.

The incidence of OSCC has been increasing because of betel nut chewing^3^. Depending on the tumor burden, radical wide excision of the tumor with or without neck dissection is the primary treatment strategy, followed by postoperative radiotherapy with or without chemotherapy^4^. This type of cancer is usually difficult to operate^5^. For medically inoperable or locally advanced unresectable disease, definitive concurrent chemoradiotherapy (CCRT) is the treatment of choice^6,7^. Despite advances in diagnosis and treatment, the local control and 5-year survival rate remain poor for patients with OSCC^8,9^. The patients taking Cisplatin following side effects, including kidney toxicity, ototoxicity hearing loss, and peripheral neuropathy. After long-term cisplatin exposure, OSCC cells would develop resistance^10^. Therefore, the Cisplatin resistance in Oral cancers becomes a major challenge and published in previous paper^11^. A subset of cancer cells gains cancer stem cells (CSCs)-like behavior during malignant progression by reactivating the complex program of epithelial-to-mesenchymal transition (EMT). EMT is mediated by several key transcription factors, which are under the control of a large range of developmental signals and extracellular cues^12^. Unraveling molecular principles that drive EMT would provide a better understanding of tumor cell plasticity and help establish new treatment modalities, including new drug targets for more effective, less toxic, and personalized therapy for patients with OSCC.

Genetic and epigenetic modifications, tyrosine kinases and their inhibitors (TKIs), and CSCs-like properties play a vital role in the treatment of OSCC^13,14^. Methods for extracting and identifying CSCs continue to evolve^15,16^. The human OSCC-derived cell lines: SAS and HSC-3 both are commonly used in the experiment of oral squamous cell carcinoma. TW2.6 cells exhibited morphological features of squamous cell carcinoma and provides a valuable model of buccal carcinoma in histological examination^17^. Side population (SP) cells are thought to be enriched in CSCs, and these are sorted based on their ability to efflux Hoechst 33342, a fluorescent DNA-binding dye^18^. These SP cells demonstrate higher proliferation, self-renewal, and radio- and chemoresistance^19,20^, thus representing an in vitro CSCs-like model and being potential targets for screening novel anticancer drugs.

Among these, the influence of protein tyrosine kinases on the oncogenicity of OSCC cannot be overemphasized^21^. Cetuximab (chimeric IgG1 monoclonal antibodies), a tyrosine kinase inhibitor of the epidermal growth factor receptor, has been used against head and neck cancers^22^. Cetuximab combined with radiotherapy improves overall survival for most head and neck cancer, but the effect is not favorable for OSCC^23^. In the GORTEC 2007-02 phase III randomized trial, it was found to be nonsuperior to traditional chemotherapeutic agents^24^. Therefore, a new treatment regimen targeting OSCC needs to be developed. Bruton’s tyrosine kinase (BTK) is a nonreceptor cytoplasmic tyrosine kinase that is primarily expressed in cells of hematopoietic lineage^25^. BTK is a crucial mediator in coupling activated immune receptors in downstream signaling pathways that affect diverse biological functions, from cellular differentiation, proliferation, and adhesion to innate and adaptive immune responses^26,27^. High-BTK expression in hematopoietic tissues plays a critical role in the differentiation of blood cells; especially, mutations in the *BTK* gene result in X-linked agammaglobulinemia in humans and X-linked immunodeficiency in mice^28^. Furthermore, BTK is actively involved in the intracellular signal transduction of G-protein-coupled receptors, lymphocyte surface antigens, cytokine receptors, toll-like receptors, and integrin molecules. Its crosstalk plays a critical role in the tumorigenesis of several malignancies^29,30^. Ibrutinib (Imbruvica, PCI-32765), a small-drug inhibitor of BTK, has been examined in several preclinical and clinical cancer studies^30–32^. Recently, a selective second-generation BTK inhibitor, acalabrutinib (ACP-196), has been explored in preclinical research^33^.

In this study, we investigated the feasibility of the BTK-mediated attenuation of OSCC cell viability, suppression of OSCC-CSCs-like attributes and associated pluripotency, deregulation of the constitutive CSCs-EMT loop in OSCC, and enhancement of OSCC cell sensitivity to concurrent chemoradiotherapy.

## Materials and methods

### Patient selection, tissue samples, cell lines, and culture

This study was approved by the Institutional Review Board of the Tri-Service General Hospital (TSGH) (IRB: 2-106-05-173) and was performed according to the recommendations of the Declaration of Helsinki for biomedical research. After patients with OSCC provided informed consent, tissue samples from TSGH were archived retrospectively. We evaluated BTK expression in 70 patients with OSCC (63 men and 7 women; age: 29–72 years, median age: 50 years) whose tissue samples were collected between January 2005 and July 2010. The tissue array was constructed using tissue samples from 70 patients with OSCC, including 52 treatment-naïve tissues and 18 CCRT-resistant recurrent OSCC tissues. Pretreatment evaluations included a detailed clinical history, physical examination, barium swallow X-ray, upper gastrointestinal tract endoscopy, and computed tomography scans of the thorax and abdomen. All patients were treated according to the standard treatment of TSGH and NCCN guidelines. Antibody against BTK (1:400, SC-81159, Santa Cruz, CA, USA) was used according to the standard immunohistochemistry (IHC) staining protocol. A similar dilution of control mouse IgG was used as a negative control. BTK expression was confirmed by two independent pathologists. The percentage distribution (P) of BTK-stained tumor cells was scored in a range of 0–100%. The intensity (I) of BTK expression was scored based on a 4-point scale (3, strong staining; 2, moderate staining; 1, weak staining; and 0, no staining). We calculated BTK immunoreactivity using the quick score (*Q*-score) method, for which the formula is *Q* = *P* × *I*; maximum = 300)^29^. We obtained the human OSCC cell line SAS (poorly differentiated human tongue SCC), TW2.6 (areca quid and tobacco-smoke-associated buccal SCC), and HSC-3 (human OSCC) kind gifts from Dr. Chi-Tai Yeh at Taipei Medical University—Shuang Ho Hospital (Taipei, Taiwan). Adherent cells were grown in RPMI-1640 medium supplemented with 10% fetal bovine serum, streptomycin (100 μg/mL), and penicillin (100 IU/mL) as a monolayer culture in a humidified 5% CO_2_ atmosphere at 37 °C and were subcultured every 48–72 h.

### Establishment of IR-resistant human OSCC cell lines

In preliminary studies to determine optimal IR dose, SAS, TW2.6 and HSC-3 cell lines were exposed to IR of 2–10 Gy for 5 consecutive days to determine the maximum tolerated dose (MTD). Based on the cell dysmorphia, cytoplasmic vacuolization, nuclei pleomorphism, and cell hyperplasia in irradiated OSCC cells compared to the control group, MTD was determined to be 2 Gy/per day for the 5 consecutive days in all three OSCC cell lines. Thus, to establish IR-resistant cell lines, the cells were subsequently exposed to 2 Gy at 130 kV, 5.0 mA, every 48 h for 30 cycles (i.e., 60 Gy cumulative dose in 2 months), using the Faxitron^®^ CellRad X-ray cell irradiator (Precision X-ray Irradiation, North Branford, CT, USA). The viable OSCC cells after the 30 IR cycles were designated IR-resistant—SAS, TW2.6 and HSC-3 Culture media was changed every 48–72 h or cells subcultured if confluent. To confirm IR-resistance, the SAS, TW2.6 and HSC-3 alongside their control counterparts were exposed to 0.5–2 Gy single-doses of IR, then evaluated using functional assays, including cell viability and clonogenic-survival assay. The OSCC-IR cell survival fractions, clonogenicity, and tumorsphere formation efficacy were markedly higher compared to the OSCC control cells.

### Drug and reagents

Cisplatin (cis-diamineplatinum (II) dichloride, #479306, 99.9% trace metal basis) was purchased from Sigma-Aldrich, Inc. (St. Louis, MO, USA). Stock solutions of 100 mM in sterile ddH2O were stored in the dark at 4 °C, respectively, until use. The stock of ibrutinib (PF-06658607, 98%, HPLC) was purchased from Sigma-Aldrich, Inc. (St. Louis, MO, USA) and prepared by dissolving 20 mg/mL of the mixture in DMSO. The stocks of each drug were stored at −20 °C until use.

### The effects of Cisplatin and ibrutinib on cell proliferation were detected using the sulforhodamine B (SRB) assay

OSCC cells were seeded in cell culture plates and treated with the drugs (Cisplatin or ibrutinib or in combination) at different concentration for 48 h, respectively. After respective drug treatments, the relative cell number was estimated by the SRB reagent according to the manufacturer’s protocol (Sigma, USA). Using the CompuSyn softwere to calculate the half-maximal inhibitory concentration (IC_50_) values of difference cell line as previously reported. The calculation method of IC_50_ is as described in the PC Software and User’s Guide on the ComboSyn Inc.website (http://www.combosyn.com). The CI was calculated using CompuSyn software. A CI value less than 1 represented synergism.

### shRNA lentivirus construction and infection

Lentivirus containing BTK short hairpin (sh) RNA was purchased from Thermo Fisher Scientific (USA) and prepared strictly as the manufacturer’s instructions. Two clones of shRNA were used to effectively knockdown BTK expression, namely A6 (shRNA1, clone ID: V2LHS-89195) and B10 (shRNA2, V3LHS-639151). shRNA lentivirus infection and construction were conducted according to standardized practice guidelines in our certified BSL-2 laboratory in The Integrated Laboratories for Translational Medicine, TSGH.

### Immunoblotting and Immunohistochemical analysis

Immunohistochemical (IHC) analysis were conducted with standard procedures. BTK protein positive signals primarily located in the cell cytoplasm, which were counted according to the brown diaminobenzidine precipitate. The purified standard BTK (C481S, Human, full-length recombinant, MW: 77 kDa, Promega, Wisconsin, USA) as loading control. Western blotting was performed using the standard method. Briefly, after preparation of whole-cell lysates, proteins were separated through sodium dodecyl sulfate-polyacrylamide gel electrophoresis and were then transferred to polyvinylidene fluoride membranes. Specific primary antibodies used were anti-BTK (1:600, sc-81159; Santa Cruz), anti-T cell immunoglobulin-3 (TIM-3) (1:1000, ab185703, rabbit; Abcam), CD133 (1:1000, ab19898, rabbit; Abcam), vimentin (1:1000, ab137321, rabbit; Abcam), and anti-phospho-Btk (Tyr223) (1:1000, #5082, rabbit mAb; Cell Signaling). Anti-GAPDH (1:10000, sc-47724, Mouse mAb; Santa Cruz) served as the loading control.

### Tumorsphere formation assay

For tumorsphere formation, we seeded 500 SAS or TW2.6 human OSCC cells in six-well ultra-low attachment plates (Corning Inc., Corning, NY, USA) containing DMEM/F12 medium supplemented with B27 (Invitrogen, Carlsbad, CA, USA), 20 ng/mL EGF (Millipore, Bedford, MA, USA), and 20 ng/mL bFGF (Invitrogen, Carlsbad, CA, USA). Tumorspheres of the first to the third generation (diameter > 50 µm) were collected and used for subsequent experiments.

### Immunofluorescence analysis

Wild-type control or BTK-knockdown (shBTK) OSCC tumorspheres were plated in six-well chamber slides for 24 h for immunofluorescence analysis. The tumorspheres were fixed with 2% paraformaldehyde and probed with primary antibodies against BTK, TIM-3, CD133, Krüppel-like factor 4 (KLF4) (1:500, ab129473, rabbit; Abcam), E-cadherin (24E10) (1:500, #3195, rabbit mAb; Cell Signaling), and vimentin. A fluorophore-conjugated secondary antibody was added to check the positive signal using a Zeiss Axiophot (Carl Zeiss) fluorescence microscope. The nuclei of viable cells were detected through 4′,6-diamidino-2-phenylindole (DAPI) staining.

### Flow cytometry

Cancers stem like cells are characterized by increased aldehyde dehydrogenase (ALDH) activity, which can be detected with Hoechst 33342 dye efflux in various cancer types. The ALDEFLUOR Kit (Stem Cell Technologies, USA) was used to determine ALDH activity in human OSCC cell lines following the manufacturer’s protocol. Briefly, after detaching SAS or TW2.6 cells from the culture dishes with trypsin/EDTA (Invitrogen, NY, USA), cells were suspended in the buffer containing an ALDH substrate and incubated at 37 °C for 1.5 h. Flow cytometry was performed using BD LSRFortessa (BD Biosciences, USA), and results were analyzed using BD software. Annexin V was used to detect drug-induced apoptosis^30^. PE-Annexin V and its binding buffer were purchased from Becton–Dickinson.

### Quantification of the effect of the treatments

For the validation step 96-well plates were used. For the assessment of the combined treatment, ALDH^+^ SAS cell line was treated simultaneously with serial concentrations of the drugs. Results were examined by isobologram analysis with Combinatorial drug interaction was evaluated and quantified by isobologram and CI values derived using CompuSyn software (CompuSyn, Inc., Paramus, NJ, USA). CI < 1, =1, and >1 indicated drugs’ synergism, additivity, and antagonism, respectively. All the experiments were done at least three times and with three replicates in each experimental group.

### Wound healing and Matrigel cell invasion assays

For the wound healing migration assay, SAS and TW2.6 cells were seeded in six-well plates and cultured until 100% confluence. Next, sterile 200-µL micropipette tips were used to create same-size scratch wounds along the median axis of the monolayer of adherent cells. The OSCC cell migration ability demonstrated by wound-gap closure was monitored over time, and photographs were taken immediately after scratch wounds were created and at indicated time points. For the Matrigel cell invasion assay, BD Falcon cell culture inserts (BD Biosciences) were used. OSCC cell suspensions were seeded in the upper surface of the insert chamber, and the lower part was filled with culture medium containing 10% fetal bovine serum. After incubation in a humidified 5% CO_2_ atmosphere at 37 °C for 24 h, the noninvaded cells in the upper part were carefully removed using sterile cotton swabs, and the invaded cells in the lower side were fix stained with 0.1% crystal violet, and counted under a microscope.

### Colony formation assay

After counting the sorted cells, 1 × 10^3^ SAS, TW2.6 or HSC-3 cells, with or without their IR-resistant counterparts were pre-exposed to indicated treatment regimen for 24 h were seeded per well in 6-well plates and incubated in 5% humidified CO_2_ incubator at 37 °C for 15 days. The colonies formed (>50 cells/colony), they were washed with 1× phosphate-buffered saline, fixed with 95% methanol for 15 min, and stained with crystal violet dye for 15 min before colony counting. The ChemiDoc-XRS imager within the QuantityOne software package (Bio-Rad, Hercules, CA, USA) was used to estimate the number and size of colonies.

### Animal studies

All the animal experiments were performed under strict compliance with the Animal Use Protocol in TSGH, National Defense Medical Center (protocol IACUC-19-014). Female 4–6-week-old NOD/SCID (NOD.CB17-*Prkdc*^scid^/NcrCrl) mice were purchased from BioLASCO Taiwan. The animal experiment is set to five mice per group. First, the tumor-initiating ability test was conducted using the tumorspheres generated from naïve SAS cells. Spheroid SAS cells were subcutaneously injected (1 × 10^6^ cells/injection) into the right flank of NOD/SCID mice. Mice that did not grow tumors will be excluded from the experiment, the mice used in this experiment all grew tumors smoothly. Second, for the drug treatment test, the subcutaneous tumor model was established using tumorspheres grown from SAS cells (1 × 10^6^ cells/100 μL/injection) in NOD/SCID mice. When the tumor became palpable, mice were randomly divided into four groups: vehicle control, cisplatin only (5 mg/kg, i.p., once a week), ibrutinib only (5 mg/kg, i.p., five times/week), and combination (ibrutinib + cisplatin). The change in the tumor burden was expressed as a fold change in mm^3^ compared with its starting volume measured using a standard caliper. Mice were humanely killed after experiment completion, and tumor biopsies were collected for further analysis. The animal studies used in this experiment did not use randomization.

### Statistical analysis

We used SPSS software v22.0 (SPSS, Chicago, IL, USA) for statistical analysis. All assays were performed at least twice in triplicate. All data in the figures are expressed as mean ± standard deviation. Comparison between two groups was performed using the *t* test. All statistical tests were two-sided, and *p* < 0.05 was considered significant. The chi-square test were used to evaluate the correlation between BTK expression and clinicopathological variables of TSGH OSCC patients. Kaplan–Meier curvs used to determine the effect of low and high-BTK expression on the overall survival of TCGA OSCC patients. Cox regression analysis was performed to determine overall survival of TSGH OSCC patients relapse from CCRT.

## Results

### BTK is aberrantly expressed in CCRT-resistant OSCC tissues and influences survival rate

**Fig. 1:**
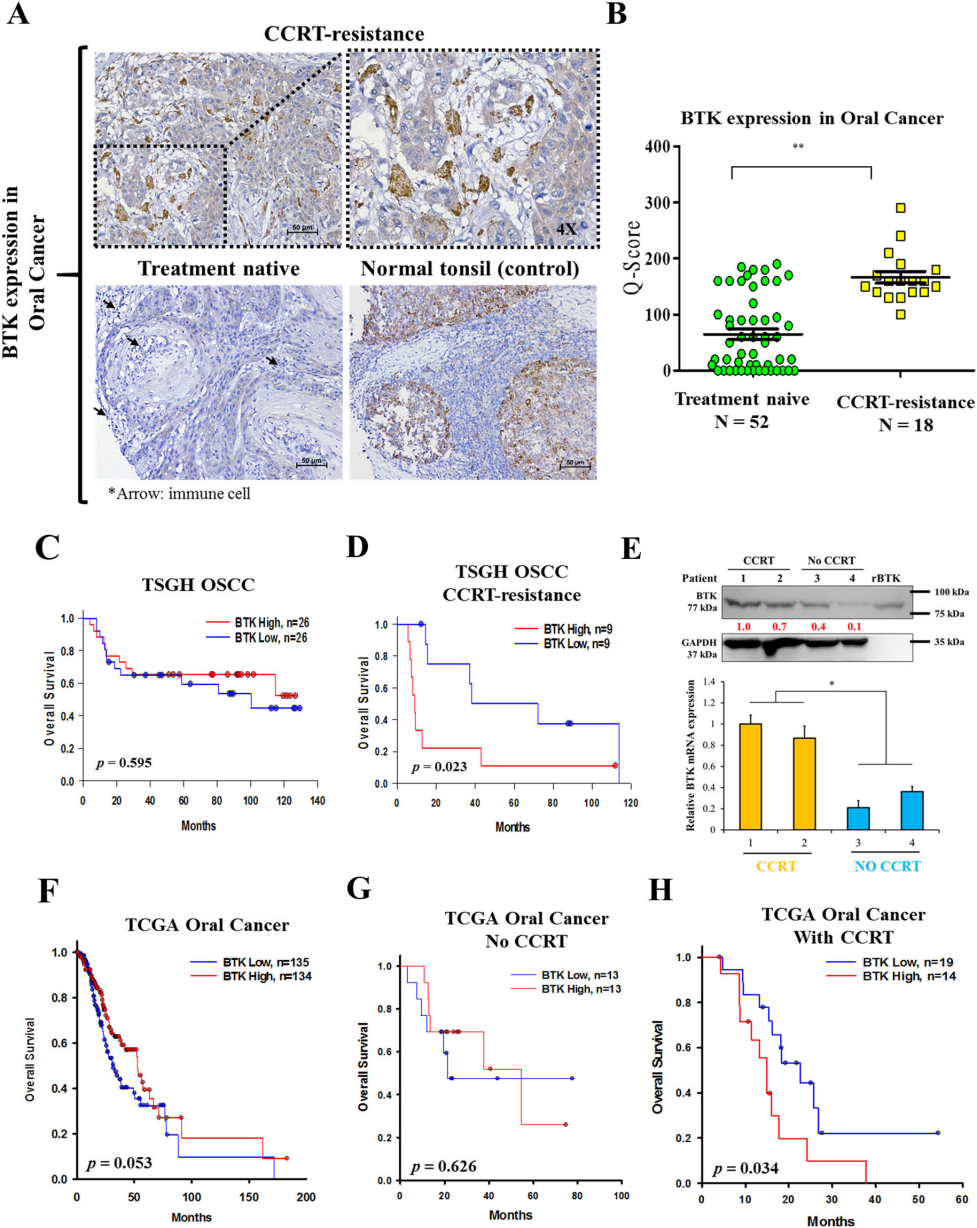
BTK is aberrantly expressed in human OSCC tissues after additional treatment and influences the survival rate. **A** IHC staining of BTK expression on OSCC tissues in CCRT-resistant (upper) and treatment-naïve tissue, normal tonsil as a control (bottom). Black arrow indicates the immune cells. **B** Q-score of BTK expression in TSGH patients with OSCC with treatment-naïve (*n* = 52) and CCRT-resistant tissues (*n* = 18). **C** Kaplan–Meier curves showing the effect of low- and high-BTK expression on the overall survival of TSGH patients with OSCC. **D** Overall survival of TSGH patients with OSCC relapses from CCRT. Median gene expression was used to determine low/high cut-off value. **E** Graphical representation of BTK expression in OSCC patients’ samples with or without additional RT/chemotherapy (top image: BTK expression in patient tissue, bottom image: BTK mRNA expression in patient tissue; rBTK: BTK recombinant protein standard, 77 kDa). **F** Kaplan–Meier curves showing the effect of low- and high-BTK expression on the overall survival of TCGA patients with OSCC (*n* = 269). **G** Overall survival of TCGA patients with OSCC without additional therapy. **H** Overall survival of TCGA patients with OSCC relapses from CCRT. Median gene expression was used to determine low/high cut-off value. *p < 0.05, **p < 0.01.

Table 1 showed the T classification and degree of differentiation of the human samples between BTK expression and clinicopathological variables of TSGH OSCC patients. A tissue array constructed using OSCC tissue samples from 70 patients was studied to determine the expression of the BTK protein using IHC staining. We show the staining of BTK in the immune cell rich areas of tumors as well as in tonsils (normal BTK level) as a control, and the immune cell areas are indicated by black arrows. BTK expression was sparsely distributed in treatment-naïve tissues, with weak intensity; by contrast, BTK expression was abundantly distributed with strong intensity in CCRT-resistant recurrent OSCC tissues (Fig. 1A). The median BTK protein expression was approximately 25% higher in CCRT-resistant tissues than in treatment-naive tissues (Fig. 1B). To explore the role of BTK in resistance OSCC, using our local TSGH OSCC cohort (*n* = 70); first, we demonstrated that high-BTK expression was associated with higher 5-year survival in the general TSGH OSCC cohort (Fig. 1C). In Fig. 1C is that patients with higher BKT expression have a better survival curve than the ones with low BKT expression, however both are nonsignificant (*p* = 0.595); then, after we separated the OSCC patients of CCRT, we were surprised to find in the TSGH CCRT-resistant OSCC cohort, patients with high-BTK expression exhibited considerably worse 5-year survival rates (Fig. 1D). After comparing the expression level of BTK in the tissues of patients, whether it is mRNA or protein, radiotherapy- or chemotherapy-treated samples exhibited higher BTK expression than their treatment-naïve counterparts (Fig. 1E). This suggests that the unusual expression of BTK after chemo- or radiotherapy might play a regulatory role in cancer cells. Moreover, consistent with our local cohort results, in the general TCGA OSCC cohort, patients with high-BTK expression exhibited higher overall survival than those with low BTK expression (Fig. 1F). However, the treatment data were missing for some TCGA OSCC cases; thus, we included only 59 cases (26 were treatment-naïve and 33 were CCRT-resistant recurrent) with complete treatment data in further analysis. TCGA OSCC patients with high-BTK expression who had not received any CCRT exhibited higher 5-year survival than those with low BTK expression (Fig. 1G). Conversely, high-BTK expression in TCGA patients with OSCC who had received CCRT was associated with significantly worse prognosis (*p* = 0.034) **(**Fig. 1H). Both TSGH and TCGA datasets revealed that the BTK expression was low in treatment-naïve tissues and high in CCRT-resistant tissues. These results suggest a role of BTK in the recurrent, treatment-resistant phenotype of OSCC cells; this informs and influences patients’ clinical outcomes; thus, BTK might is a potential histological biomarker and a prognosticator for survival in recurrent OSCC.

**Table 1:** Correlation between BTK expression and clinicopathological variables of TSGH-OSCC patients (*n* = 70).

Clinicopathological variables	No.	BTK			
		High expression	Low expression	*x*^2^	*p* Value
*Age, years*					
≦47	46	11	35	29.055	<0.001
å 47	24	22	2		
*Gender*					
Male	63	29	34	0.312	0.576
Female	7	4	3		
*T classification*					
T1–T2	46	17	29	4.147	0.042
T3–T4	24	15	9		
*N classification*					
N0	36	16	20	0.048	0.826
N1–N3	34	16	18		
*AJCC stage*					
I–II	24	7	17	4.030	0.045
III–VI	46	25	21		
*CCRT*					
Yes	18	18	0	16.154	<0.001
No	52	24	28

### Correlation of BTK with CSCs-like phenotypes of human OSCC cell lines

**Fig. 2:**
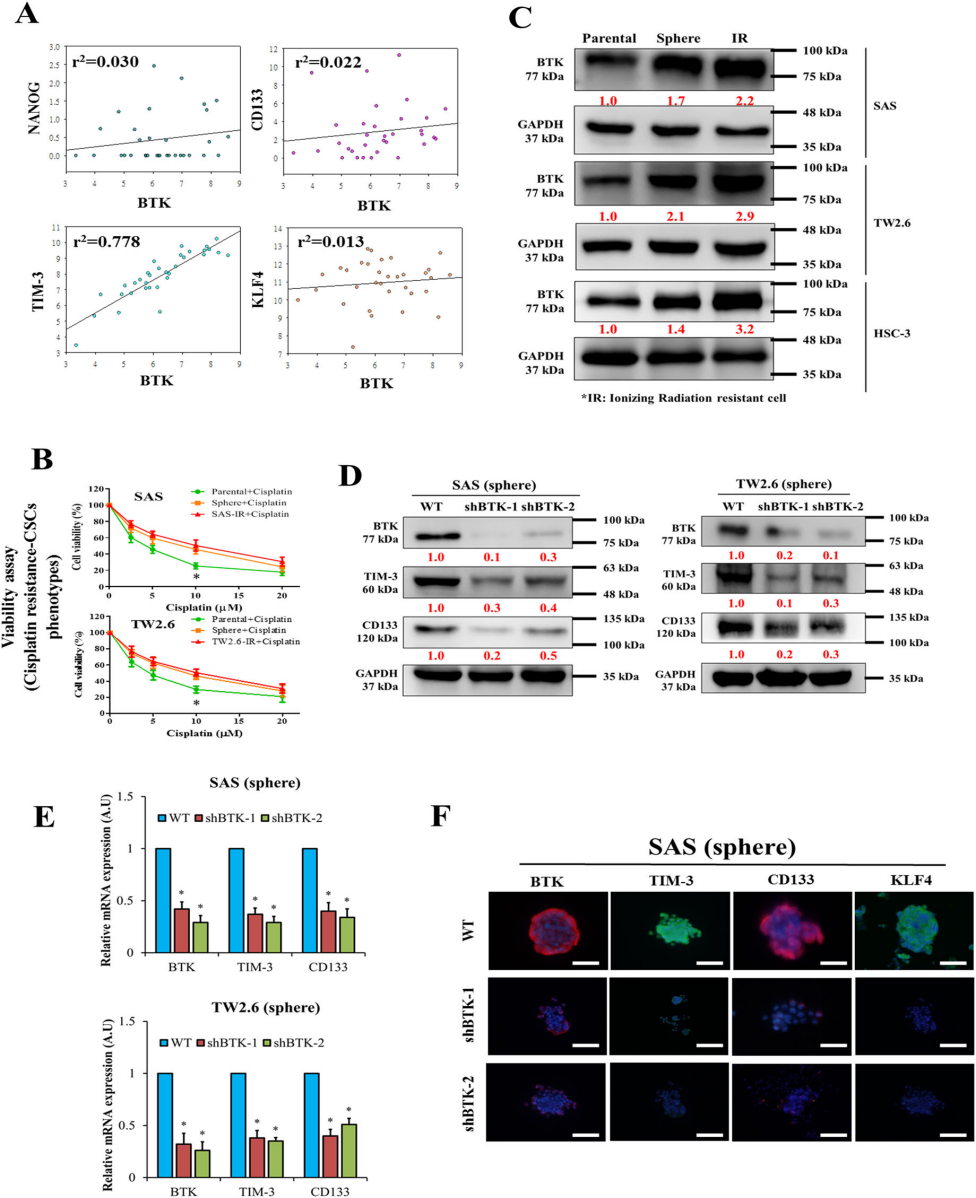
BTK is positively correlated with radiation-resistant and CSCs-like phenotypes of OSCC. **A** Scatter plot showing the correlation between the expressions of BTK mRNA and NANOG, CD133, TIM-3, and KLF4. **B** Cell viability assay revealed higher cisplatin resistance, indicated by sphere and ionizing radiation (IR)-resistant SAS and TW2.6 cells compared with the parental cells. **C** The protein BTK expression in SAS, TW2.6 and HSC-3 parental, sphere, and IR cell lines as shown by Western blotting. **D E** Effect of BTK-knockdown on BTK, TIM-3, and CD133 expressions at both mRNA and protein levels. **F** Immunofluorescence staining showing the effect of shBTK on the BTK expression, TIM-3, CD133, and KLF4 proteins in spheres formed by SAS cells. Blue stain = DAPI, nuclear staining. GAPDH is loading control; WT, wild-type. All assays were performed at least twice in triplicate and expressed as mean ± SD. *p < 0.05, **p < 0.01.

We next investigated the association between BTK and OSCC-CSCs-like phenotypes using TCGA OSCC data and found varying degrees of positive correlations between BTK mRNA and Nanog (*r*^2^ = 0.030, *p* < 0.05), CD133 (*r*^2^ = 0.022, *p* < 0.05), TIM-3 (*r*^2^ = 0.778, *p* < 0.001), and KLF4 (*r*^2^ = 0.013, *p* < 0.05) (Fig. 2A). Given that the CSCs phenotype has been broadly implicated in cancer therapy resistance^12^, we investigated links between BTK expression, CSCs-like behavior, and cisplatin resistance. We demonstrated that the OSCC tumorspheres and IR cells were significantly less responsive to treatment with 5–20 μM cisplatin than parental cells (Fig. 2B). BTK expression was markedly upregulated in the tumorspheres and IR cells (Fig. 2C). Moreover, we also perform western blot analysis loading the purified 77 kDa BTK to determine which isoform is overexpressed in OSCC cell lines. The results showed that the molecular weight of BTK overexpressed by the OSCC cell lines was close to the purified 77 kDa BTK (Supplementary Fig. S1). Furthermore, we examined the effects of shBTK on the differentiation status and self-renewal capacity of SAS and TW2.6 cells. We found reduced BTK expression in shBTK-1 and shBTK-2 SAS tumorspheres, along with a significant downregulation of TIM-3, and CD133 in shBTK-treated cells both at mRNA and protein levels compared with their wild-type counterparts (Fig. 2D, E). We further examined the effects of shBTK on these SAS-derived tumorspheres using immunofluorescence analysis, and the results demonstrated that compared with wild-type cells, shBTK significantly decreased the immunoreactivity of the stemness makers TIM-3, CD133, and KLF4 (Fig. 2F). These findings at least partly indicate that is a direct correlation between BTK levels and the expression of stemness markers.

### BTK expression regulates viability and metastasis of OSCC cells

**Fig. 3:**
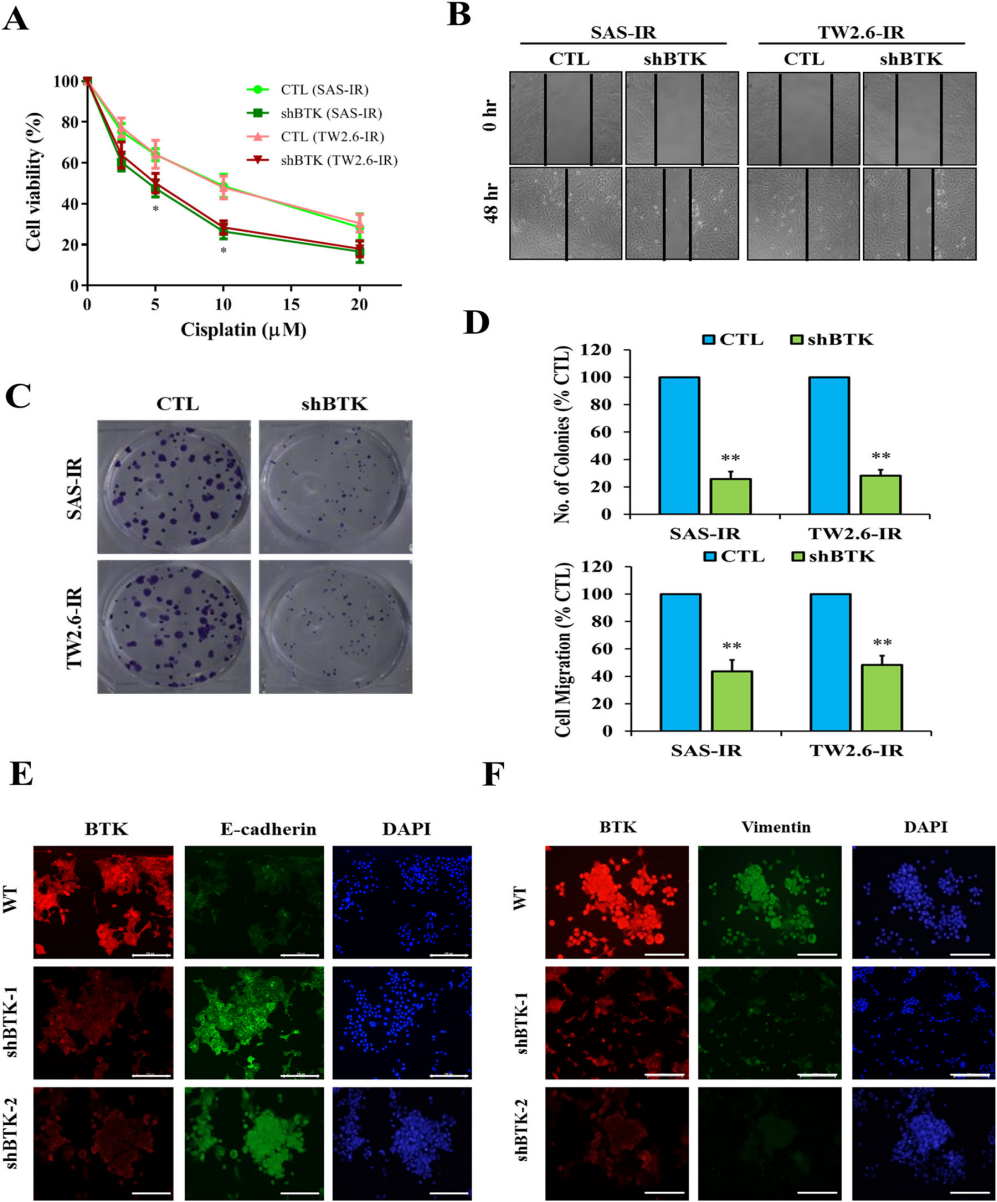
BTK expression regulates viability and the metastatic trait of OSCC cells. **A** Cell viability curve for the SAS/-IR and TW2.6/-IR cell line at 48 h of treatment with dose-dependent of cisplatin. **B** BTK-KD cells exhibited slower area closure than WT cells in the in vitro scratch wound cell migration assay after 48 h. **C** BTK-knockdown significantly decreased colony-forming abilities. **D** The quantitative data of the two types of cells. **E**, **F** Immunofluorescence staining images showing the BTK expression, E-cadherin, and vimentin in SAS-IR WT and SAS-IR cells transfected with shBTK. DAPI, nuclear staining. All assays were performed at least twice in triplicate and expressed as mean ± SD. *p < 0.05, **p < 0.01.

Next, we examined the effect of shBTK on the viability and motility of SAS and TW2.6 cells. The results of the SRB assay revealed that shBTK significantly improved chemosensitization and inhibited the viability of SAS-IR or TW2.6-IR cells treated with 0–20 μM cisplatin for 48 h (Fig. 3A). We also demonstrated that compared with wild-type control OSCC cells, shBTK significantly attenuated the migration of SAS-IR and TW2.6-IR cells (Fig. 3B) and, consistently, markedly impaired the ability of the OSCC cells to form colonies, as demonstrated by a 55% (*p* < 0.01) or 52% (*p* < 0.01%) reduction in the number of colonies formed by shBTK-treated SAS-IR and TW2.6-IR cells, respectively (Fig. 3C). The quantitative data of the two types of cells are shown in Fig. 3D. Furthermore, using the immunofluorescence assay, we demonstrated that concomitant with decreased BTK expression, shBTK caused a significant increase in E-cadherin (Fig. 3E) and downregulated vimentin (Fig. 3F). These results suggest that BTK modulates the expression and subcellular localization of EMT factors in OSCC cells and indicates a role of BTK in the EMT and metastatic phenotype of OSCC cells.

### Ibrutinib targets the ALDH-enriched radioresistant human OSCC cell population by significantly suppressing BTK expression or activity

**Fig. 4:**
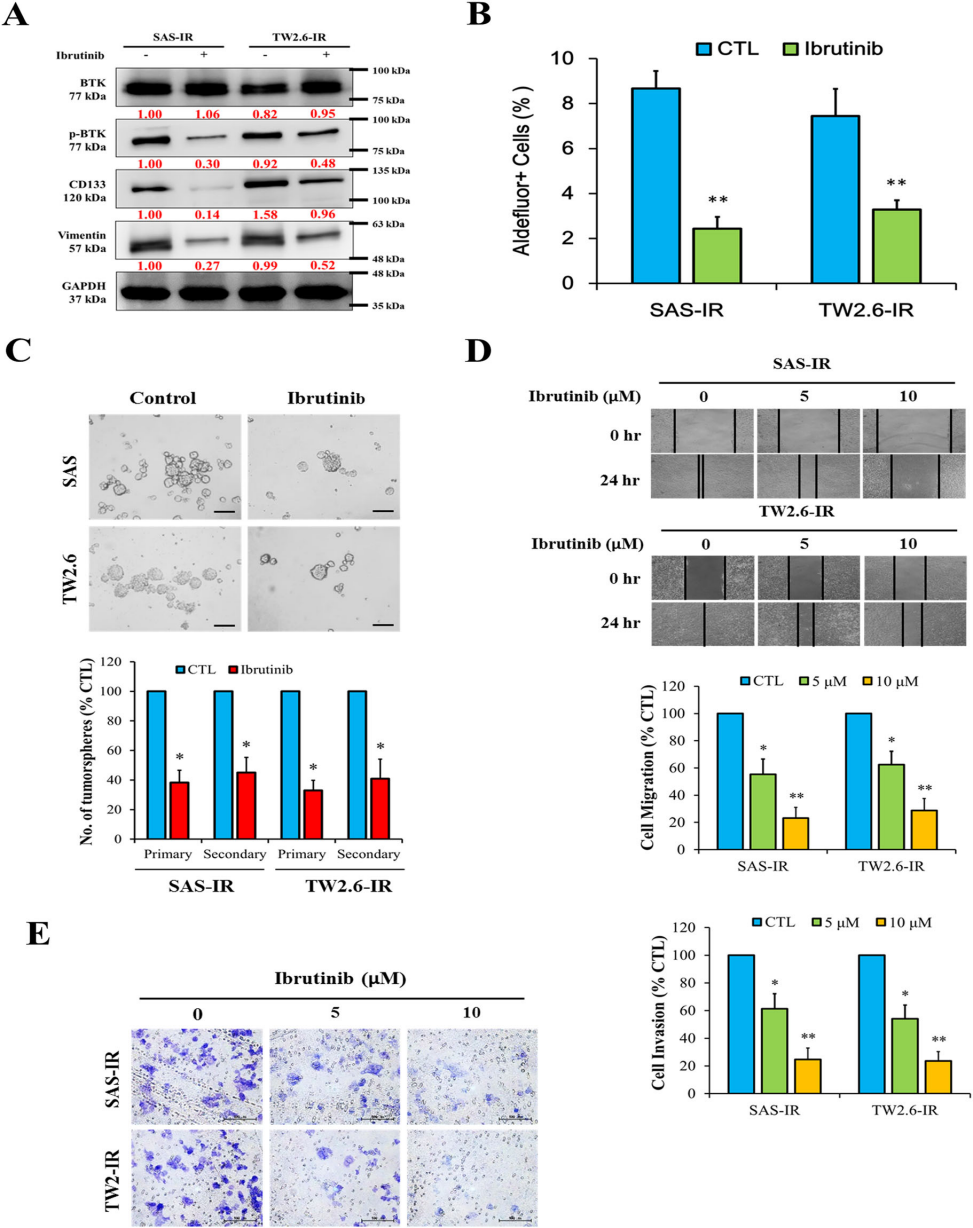
The CSCs-like trait of ALDH-rich oral cancer cell lines is significantly suppressed by ibrutinib. **A** Western blot indicated the expression levels of BTK, pBTK, stemness marker CD133, and EMT marker vimentin in SAS-IR and TW2.6-IR cell lines. GAPDH was used as a loading control. **B** Representative data showing the effect of pre-exposing SAS-IR and TW2.6-IR cells to 0 μM (control) or 10 μM (treated) ibrutinib for 48 h, on their ALDH activity as detected by flow cytometry-based ALDEFLUOR assay and the graphical quantitative analysis of treated ALDH^+^ compared with control cells. **C** The reducing effect of ibrutinib pretreatment on OSCC tumorsphere formation from SAS and TW2.6 (*left*), and quantitative representation of the effect of ibrutinib treatment on primary and secondary tumorsphere formation from SAS and TW2.6 cells (*right*). **D**, **E** Representative images illustrating that treatment with ibrutinib significantly reduced the migration and invasion ability of SAS-IR and TW2.6-IR OSCC cells in a dose-dependent manner. Graphical quantitative data showing the relative number of cells in ibrutinib-treated cells compared with control cells. All experiments performed in triplicates and expressed as mean ± SD. *p < 0.05, **p < 0.01.

We also demonstrated that treatment with ibrutinib, a first-in-class BTK inhibitor, significantly inhibited phospho-BTK protein expression levels and downregulated the stemness marker CD133 and the EMT marker vimentin in SAS-IR and TW2.6-IR OSCC cells compared with their wild-type control counterparts (Fig. 4A). Furthermore, we examined the effect of ibrutinib on ALDH activity using flow cytometry-based ALDEFLUOR assay with Hoechst 33342 dye efflux and demonstrated that the treatment of ALDH-rich OSCC cells with 10 μM ibrutinib for 48 h led to a 4.24% reduction (*p* < 0.01) in the ALDH-rich SAS cell population (8.67% in control cells vs. 2.43% in ibrutinib-treated cells) (Fig. 4B). Similarly, ibrutinib treatment reduced the population of ALDH-rich TW2.6 cells from 7.45% in the control group to 3.28% in ibrutinib-treated cells (Fig. 4B). Moreover, we examined the probable effect of ibrutinib on the self-renewal ability of OSCC-CSCs using primary and secondary SAS or TW2.6 tumorspheres, and the results demonstrated that ibrutinib caused significant quantitative and qualitative inhibition of both primary (SAS: 60% reduction, *p* < 0.05; TW2.6: 64% reduction, *p* < 0.05) and secondary (SAS: 71% reduction, *p* < 0.05; TW2.6: 60% reduction, *p* < 0.05) tumorsphere formation compared with their control counterparts (Fig. 4C). In other experiments, we assessed the effect of 5 and 10 µM ibrutinib treatment on SAS and TW2.6 cell motility. Ibrutinib treatment significantly and dose-dependently attenuated the ability of OSCC cells to migrate into the denuded area over 24 h (*p* < 0.05) (Fig. 4D). We also observed a significant reduction in the number of invaded ibrutinib-treated SAS-IR or TW2.6-IR cells compared with control cells (*p* < 0.05) (Fig. 4E). Thus, ibrutinib dose dependently induced tumorsuppression and reduced the tumor invasion ability of OSCC cells.

### Ibrutinib enhances cisplatin sensitivity in human OSCC SP-derived cells

**Fig. 5:**
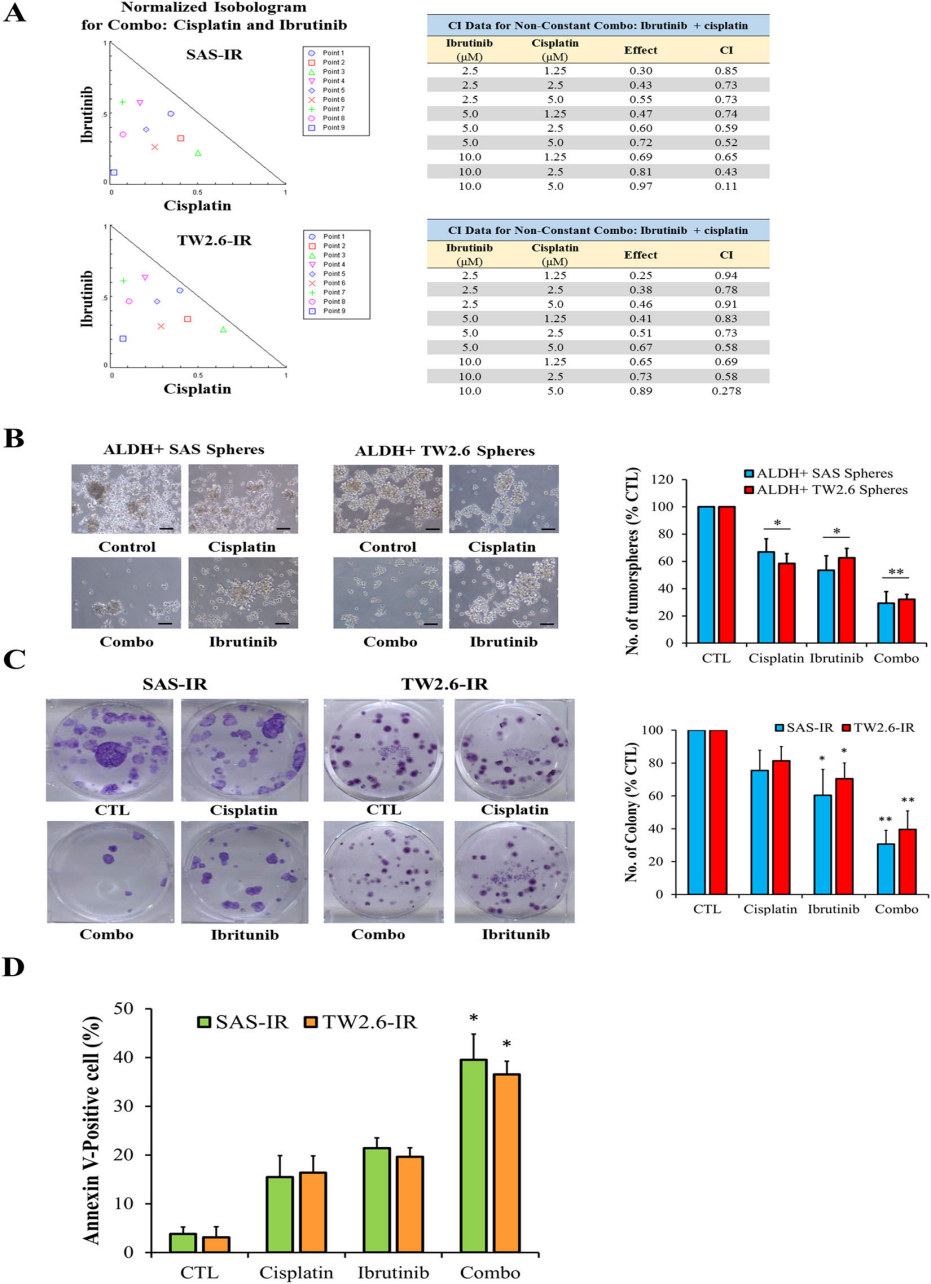
Ibrutinib reduced cisplatin-induced oral CSCs enrichment, increases apoptosis, limits the colony-forming potential, and enhances the sensitivity of the ALDH^+^ OSCC cells to cisplatin. **A** Drug combination assay: different concentrations of ibrutinib and cisplatin were used in combination for calculating the combination index (CI). Normalized isobolograms demonstrated that ibrutinib and cisplatin synergistically suppressed the cell viability of OSCC spheres. CI < 1 denotes synergy. **B** Photo images (*left*) and graphical quantitative data (*right*) of the tumorsphere-forming ability of the ALDH^+^ SAS or TW2.6 cells after treatment with 5 μM cisplatin, 5 μM ibrutinib, or both compared with control cells. **C** Treatment of ibrutinib alone or in combination with cisplatin effectively reduces the colony-forming abilities, graphical quantitative data of the number of colonies formed by the SAS-IR or TW2.6-IR cells after treatment with 5 μM cisplatin, 5 μM ibrutinib, or both compared with control cells. **D** Apoptotic effect of cisplatin alone, ibrutinib alone, or cisplatin–ibrutinib combination treatment in SAS-IR or TW2.6-IR cells. Data represent experiments performed in triplicates and expressed as mean ± SD. *p < 0.05, **p < 0.01.

We next examined if ibrutinib enhances the cytotoxic effect of cisplatin against OSCC SP cells by conducting drug combination assays with varying concentrations and the combination of ibrutinib and cisplatin. Isobologram analysis demonstrated that ibrutinib and cisplatin synergistically suppressed the viability of ALDH^+^ SAS tumorspheres, with the combination index (CI) of <1 denoting drug synergy (Fig. 5A). The previous condition of 10 µM ibrutinib was based on IC_50_ and under the synergistic effect of drugs, the concentration might be reduced to combine the two drugs to observe their effects. Although the combination of 10 µM ibrutinib and 5 µM cisplatin has the best effect, the high concentration inhibits most of the cell growth, which is not conducive to detailed observation of the experiment (SAS-IR: 0.97 and TW 2.6-IR: 0.89). Therefore, the follow-up experiment uses a combination of 5 µM ibrutinib and 5 µM cisplatin as condition. In addition, compared with control cells, we demonstrated that treatment with ibrutinib (49% reduction vs. CTL, *p* < 0.05) or cisplatin (32% reduction vs. CTL, *p* > 0.05) alone moderately reduced the ability of the ALDH^+^ SAS cells to form tumorspheres, and the inhibitory effect of ibrutinib + cisplatin was significantly stronger than that of monotherapy or control (74% reduction vs. CTL, *p* < 0.01), as evidenced by the formation of considerably smaller and fewer ALDH^+^ SAS tumorspheres (*p* < 0.05) (Fig. 5B). Similarly, treatment of SAS-IR or TW2.6-IR cells with 5 µM ibrutinib, 5 µM cisplatin, or both significantly decreased the number of colonies formed, with the ibrutinib/cisplatin combination eliciting the most inhibitory effect (*p* < 0.05) (Fig. 5C). Using the Annexin V flow cytometry assay for evaluation of drug-induced cell death, we also demonstrated a significant increase in the Annexin V-positive population of SAS-IR and TW2.6-IR cells treated with cisplatin, ibrutinib, or both, with the highest population in the combination therapy group (*p* < 0.05) (Fig. 5D).

### Ibrutinib addition enhances sensitivity to Cisplatin and improve the therapeutic response of OSCC in vivo

**Fig. 6:**
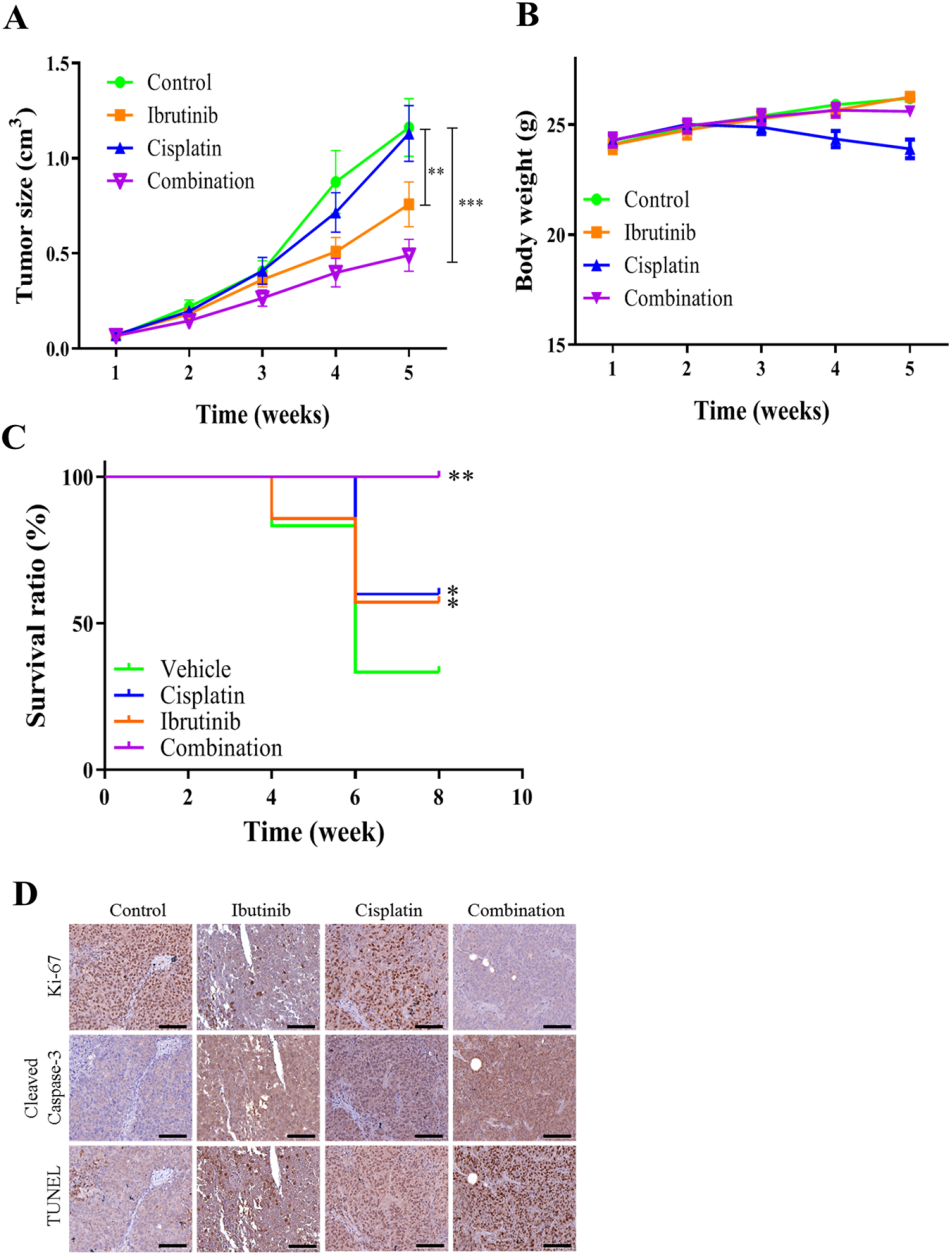
Ibrutinib improve the cisplatin-resistant SAS CSCs in vivo. **A** Photographs of oral cancer cells, SAS-IR (1 × 10^6^ cells/injection, subcutaneous) were injected into the NOD/SCID (NOD.CB17-*Prkdc*^scid^/NcrCrl) mice for establishing the tumor xenograft model (*n* = 5 per group). When tumor size became palpable, mice were separated into four groups: vehicle control, ibrutinib (10 mg/kg, i.p., 5 times a week), cisplatin (5 mg/kg, i.p., once a week), and ibrutinib + cisplatin. **B** Tumor volume versus time effect curve. Ibrutinib + cisplatin treatment displayed the most significant decrease in tumor volume followed by ibrutinib-only treatment, whereas cisplatin only and vehicle treatments displayed the largest tumor volume. **C** Kaplan–Meier survival curve. Mice receiving the ibrutinib + cisplatin regimen exhibited the highest survival ratio followed by the ibrutinib only group, whereas the cisplatin only and vehicle groups exhibited the lowest survival ratios. **D** IHC image of of the xenograft tumors revealed that the ibrutinib/cisplatin combination effectively inhibited the expression of Ki-67 and induction of cleaved capase-3. *p < 0.05; **p < 0.01; ***p < 0.001. Scale bar: 100 μm.

Finally, we evaluated the anticancer effect of ibrutinib in murine OSCC tumor xenograft models in vivo. Consistent with in vitro findings, we found that cisplatin, ibrutinib, and ibrutinib/cisplatin combination caused mild, moderate, and strong suppression of tumor growth, respectively, as evidenced by a 0.04 cm^3^, 0.47 cm^3^ (*p* < 0.01), and 0.72 cm^3^ (*p* < 0.001) reduction in the average volume of tumors grown in mice, respectively, by week 5 (Fig. 6A). Moreover, although no apparent change in the average body weight was noted in the control, ibrutinib alone, or ibrutinib/cisplatin combination groups, cisplatin-treated mice exhibited an average body weight loss of 4.15 g, (Fig. 6B). Furthermore, survival analyses indicated that compared with the 100% survival of mice in the combination group by week 8, cisplatin- and ibrutinib-treated mice exhibited 64% and 60% survival, respectively, whereas survival was only 33% in vehicle-treated mice (Fig. 6C). Consistent with the in vitro data, immunohistochemistry and terminal deoxynucleotidyl transferase dUTP nick end labeling (TUNEL) analyses (Fig. 6D) of the xenograft tumors revealed that the ibrutinib/cisplatin combination effectively inhibited the tumor proliferation marker Ki-67. Moreover, ibrutinib facilitated cisplatin-induced apoptosis in OSCC xenograft tumors, as evaluated using cleaved caspase-3 staining and the TUNEL assay.

## Discussion

OSCC is one of the most diagnosed and lethal head and neck cancers worldwide. The current treatment goal of OSCC is to enhance therapeutic response, decrease side effects, and improve esthetics using a combination of radical surgery, radiotherapy, and chemotherapy^6–8^. Multimodality treatment, including surgery followed by CCRT, in the locally advanced stage is necessary; however, several randomized clinical trials have demonstrated that the treatment response remains limited. Cisplatin alone does not improve overall survival because of poor tumor response, and it is associated with chemotherapy-induced nausea and vomiting as well as other harmful adverse effects such as hearing impairment and nephrotoxicity^4,7^. Nevertheless, cisplatin remains the first-line chemotherapeutic agent for OSCC despite its limited tumor response and severe side effects. We investigated a strategy to enhance the tumor-killing effect of cisplatin while reducing its dose and, consequently, adverse effects. There are more evidences showing CCRT-resistance in squamous cell carcinoma patients^34^. The possible reason is CSCs which have the drug-resistance and lead to tumor relapse.

The molecular mechanism underlying the anticancer effect of cisplatin involves cross-linking with target DNA, interfering with DNA replication and then inducing mitochondrial apoptosis. However, cancer cells evade cisplatin-related cellular death through many mechanisms^35^. One of the common mechanisms is increased platinum (cisplatin) efflux through relatively nonselective members of the ATP-binding cassette (ABC) transporters, thus decreasing cisplatin accumulation in the cells. CSCs overexpress ABC transporters, resulting in increased platinum efflux, and thus, they have cancer initiation and self-renewal abilities in OSCC^36^. CSCs, which we obtained from ALDH^+^ SPs by using flow cytometry-aided cell sorting, comprise a useful tool to evaluate cancer treatment responses^15,16^. Overexpression of ALDH activity in CSCs is significantly correlated with poor clinical outcomes in breast and ovarian cancers^37^. In head and neck cancers, CSCs-like SP cells are highly resistant to cisplatin treatment and are strongly associated with poor survival^32,38–40^. In this study, we explored the prognostic role of BTK in CCRT-resistant cancer cells, and we hypothesized that BTK exhibits a strong positive correlation with the stemness regulator genes, and that ibrutinib, a first-in-class small molecule inhibitor of BTK, can be combined with cisplatin to effectively inhibit ALDH^+^ OSCC-CSCs. Tumor-associated macrophages (TAM) can promote the initiation and metastasis of tumor cells. The TAM receptors (Tyro3, Axl, and MerTK) are a unique family of tyrosine kinase receptors, which have become increasingly important, with a potential role in the era of targeted therapy^41^. Ibrutinib is able to inhibit BTK phosphorylation in TAM generated in vitro and impaired the ability of these cells to produce IL-1β^42^.

First, we demonstrated that BTK was highly expressed in CCRT-resistant OSCC tissues and influenced clinical survival, as evidenced by IHC staining of the TSGH OSCC tissue array, compared with treatment-naïve cancer tissue (Fig. 1A). We also used the *Q*-score method to represent the quantitative difference in BTK expression between treatment-naïve and CCRT-resistant cancer tissues (Fig. 1B). The OSCC database from TCGA revealed that radioresistant or chemoresistant cancer cells highly expressed the *BTK* gene (Fig. 1D). For patients newly diagnosed with OSCC, *BTK* gene expression did not significantly influence the overall survival; however, it significantly influenced the overall survival in CCRT-resistant relapse cancer patients (Fig. 1E, F). From the results of the TCGA data set, we can infer the possible reasons for the high expression of BTK. Although the expression of BTK may be affected by the number of immune cells, it is considered that after chemotherapy and radiotherapy will cause the decline of immune cells, we speculate that BTK here may be highly expressed by CCRT-resistance OSCC cancer cells. We supposed that CCRT-resistant cancer cells, which might be CSCs, expressed BTK and influenced patient survival^40^. The high expression BTK level in myeloma cells increased features of cancer stemness might leads to dependent upregulation of key stemness genes (OCT4, SOX2, NANOG, and MYC)^43^.

We then provided evidence of the positive correlation between increased BTK expression and CSCs-like phenotype of OSCC cells. Using the TCGA OSCC cohort data, we demonstrated that BTK was positively correlated with the stemness regulator genes Nanog, CD133, TIM-3, and KLF4 (Fig. 2A). Tumorsphere formation is a technique in which CSCs are enriched in various tumors. A tumorsphere is an in vitro CSCs model and is characterized by self-renewal and enhanced proliferative abilities^44^. Tumorspheres are common features of human OSCC primary cultures^45^. Pharmacological targeting of OSCC-CSCs is a promising platform for preclinical drug testing^16,40^. In the present study, we used two common human OSCC cell lines: SAS and TW 2.6. SAS cells originate from poorly differentiated tongue cancer of very aggressive nature. The TW2.6 cell line was established from a buccal mucosa cancer patient and provides a model for betel-nut-chewing-and-tobacco-smoke-associated buccal carcinoma^46^. Both types of cancer represent the most common OSCC in Taiwan (Taiwan Cancer Registry. Health Promotion Administration, Ministry of Health and Welfare, http://tcr.cph.ntu.edu.tw/main.php?Page=A1). We also established a link between high-BTK expression and the CSCs-like and cisplatin resistance phenotype of OSCC (SAS and TW2.6) cells compared with parental cells (Fig. 2B, C). However, shBTK downregulated the aforementioned stemness regulator markers in tumorspheres (Fig. 2D, E), suppressed cell viability, promoted drug sensitivity, and attenuated OSCC cell migration (Fig. 3A, B). BTK knockdown reduced the number of colony/tumorsphere formation, stemness of OSCC cells, and the EMT markers—E-cadherin and vimentin (Fig. 3D, E).

There are different isoforms of the Bruton tyrosine kinase described so far. According to GenBank, BTK has five isoforms: isoform 1 (NP_000052), isoform 2 (NP_001274274), isoform 3 (NP_001274273), BTK truncate form (KF241986) and P65BTK with corresponding molecular weight of 76, 57, 80, 65.5, and 65 kDa^47^. The 77 kDa isoform originally identified for its being mutated in XLA patients and mainly expressed in bone marrow-derived cells has been been recently shown expressed also in neuroblastoma and esophageal cancer^48,49^. An 80 kDa has been described in breast and prostate cancer^50,51^, and a 65 kDa isoform has been reported in colon cancer, glioblastoma and NSCLC^47,52,53^. Even though they are all inhibited by Ibrutinib, given that the kinase domain in conserved in all the isoforms, it is important to determine which isoform is overexpressed in OSCC since they are regulated differently and enter in different signaling pathways. BTK is involved in transducing activation, proliferation, maturation, differentiation and survival signals and is an upstream activator of multiple antiapoptotic signaling molecules and networks. According to the results of Western blotting, compared with the purified standard BTK (C481S, Human, full-length recombinant, MW: 77 kDa, Promega, Wisconsin, USA), the molecular weight of endogenous BTK is close to 77 kDa.

Combining the previously reported biochemical activity of ibrutinib against EGFR and the fact that similar reactive cysteine residues are located at the same positions in EGFR and BTK^54^, we performed a comprehensive comparison study of ibrutinib reduces the expression of EGFR phosphorylation in OSCC cells (Supplementary Fig. S2). In radiation-tolerant OSCC cells, BTK may be involved in the EGFR/AKT/mTOR signaling pathway and increase the drug sensitivity of cancer cells after radiotherapy. Ibrutinib combined with radiation induced G2/M arrest and cell apoptosis. Ibrutinib decreased the phosphorylation of EGFR, then reversed the upregulation of p-AKT and downstream genes by radiation^54^.

Our Western blot analyses confirmed that ibrutinib efficiently targeted and reduced pBTK protein expression (Fig. 4A) by creating a covalent bond with the BTK cysteine-481 residue and then inhibiting the BTK activity. Several preclinical findings indicate that ibrutinib induces apoptosis and impairs cancer cell proliferation. Ibrutinib has been tested in clinical hematological diseases including leukemia, lymphoma, and Waldenström macroglobulinemia in phase 1/2 trials^30,31^. Furthermore, some preclinical studies have revealed that ibrutinib can inhibit the growth of solid tumors, such as skin, lung, breast, and ovarian cancers^32,44,55,56^. Using the flow cytometry-based ALDEFLUOR assay, we demonstrated that ibrutinib reduced the ALDH-rich OSCC cells (Fig. 4B). Ibrutinib reduced tumorsphere formation of SAS and TW2.6 cells and influenced the size, quality, quantity, and tumor appearance (Fig. 4C). The tumor migration and invasion abilities were dose-dependently inhibited by ibrutinib (Fig. 4D, E). In clinical practice, cisplatin remains the standard of care for patients with OSCC; however, the response rate is limited^4,6,7^. Furthermore, CSCs-enriched radio- or chemoresistant cancer cells constitute a therapeutic enigma, being nonresponsive to cisplatin initiation or re-challenge^40,57^. ALDH^+^ OSCC cancer cells are inherently CSCs and were used for the drug test. Both ibrutinib and cisplatin reduced ALDH^+^ OSCC tumorsphere formation, but the inhibitory effect was optimal when they were administered together (Fig. 5A, B). Both the colony formation assay and Annexin V flow cytometry proved that the ibrutinib/cisplatin combination achieved the optimal tumor-killing effect for ALDH^+^ OSCC cells (Fig. 5C, D).

Finally, we evaluated the therapeutic role of ibrutinib using the SAS-IR CSCs (cisplatin-resistant) xenograft model. The ibrutinib/cisplatin combination led to the first largest reduction in the tumor burden, with ibrutinib monotherapy having the second largest reduction. Notably, ibrutinib caused significant tumor growth inhibition in vivo and re-sensitized cisplatin-resistant tumors to cisplatin treatment (Fig. 6). We also demonstrated that shBTK downregulated the immune checkpoint receptor TIM-3. This is essential because TIM-3 is selectively expressed on CD4^+^ T helper 1 and CD8^+^ T cytotoxic cells^58^, and its expression on T cells activates myeloid-derived suppressor cells, which subsequently suppress intratumoral immune responses^59–61^. Taken together, these findings indicate that silencing BTK at least partly promotes cancer immune response by targeting TIM-3 signaling.

**Fig. 7:**
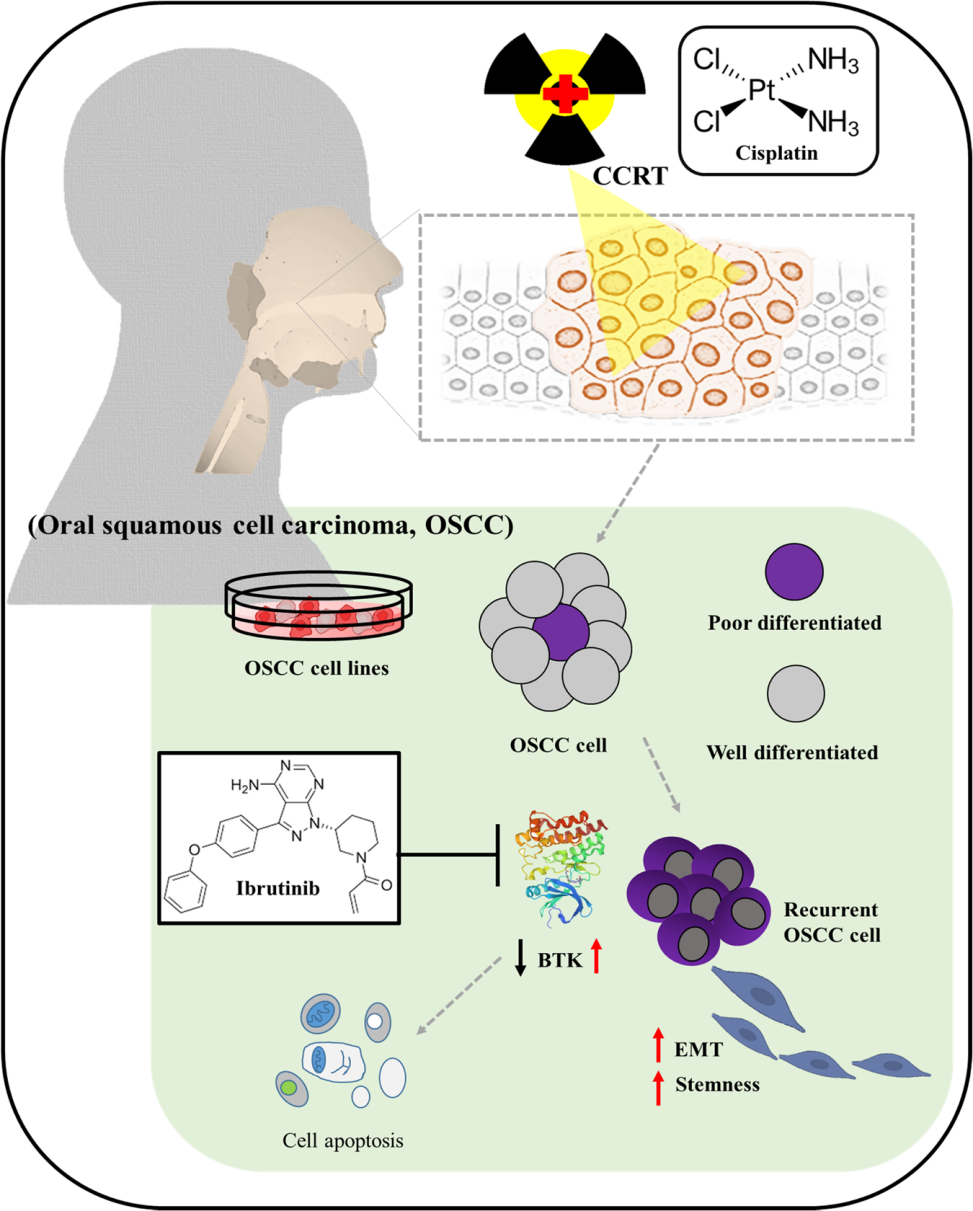
Graphical abstract. A pictorial abstract showing BTK-related molecular network and how the suppression of the integrin-associated signal transducer BTK enhances radio- or chemosensitivity by downregulating CSCs-associated pluripotency factors and deactivating EMT in OSCC cells.

To the best of our knowledge, the present study is the first to demonstrate the therapeutic efficacy of targeting BTK in CCRT-resistant OSCC. BTK expression in CCRT-resistant OSCC may be a useful histological biomarker. The limitations of this study include the short follow-up and the small number of CCRT-resistance patients, which make comparisons between treatment groups difficult. Importantly, we provide the first preclinical evidence that the suppression of the integrin-associated signal transducer BTK enhanced radio- or chemosensitivity by downregulating CSC-associated pluripotency factors TIM-3, Nanog, and KLF4 and by deactivating EMT in OSCC (Fig. 7). Ibrutinib reduced BTK, suppressed CSCs-like traits, reversed CCRT-resistance, and enhanced the tumor-killing effect of cisplatin against OSCC cells.

## Supplementary information

Supplementary Information

Conflict of Interest Statement

Graphical Abstract

Highlights
