# Supplementary material for: Inhibition of Bruton’s tyrosine kinase as a therapeutic strategy for chemoresistant oral squamous cell carcinoma and potential suppression of cancer stemness
Source: Oncogenesis. 2021 Feb 27;10(2):20. doi: 10.1038/s41389-021-00308-z (PMC7914253; doi:10.1038/s41389-021-00308-z)
Supplement: Supplementary file 1 — Supplementary Information [file 41389_2021_308_MOESM1_ESM.docx]

**Supplementary Materials**

**Inhibition of Bruton’s tyrosine kinase as a therapeutic strategy for chemoresistant oral squamous cell carcinoma and potential suppression of cancer stemness**

Shao-Cheng Liu^1#^, Yang-Che Wu^2,3#^, Chih-Ming Huang^4#^, Ming-Shou Hsieh^2,3^, Ting-Yi Huang^5,6^, Chin-Sheng Huang ^2,3^, Tung-Nien Hsu^2,3^, Mao-Suan Huang^2,3^, Wei-Hwa Lee^6,7^, Chi-Tai Yeh^6,7,8#^, Chun-Shu Lin^9*^

^1^ Department of Otolaryngology-Head and Neck Surgery, Tri-Service General Hospital, National Defense Medical Center, Taipei City, 114, Taiwan;

^2^ School of Dentistry, College of Oral Medicine, Taipei Medical University, Taipei City 110, Taiwan;

^3^ Department of Dentistry, Taipei Medical University - Shuang Ho Hospital, New Taipei City, 235, Taiwan;

^4^ Department of Otolaryngology, Taitung Mackay Memorial Hospital, Taiwan

^5^ Department of Hematology and Oncology, Cancer Center, Taipei Medical University - Shuang Ho Hospital, New Taipei City, 235, Taiwan;

^6^ Department of Medical Research & Education, Taipei Medical University - Shuang Ho Hospital, New Taipei City, 235, Taiwan;

^7^ Department of Pathology, Taipei Medical University-Shuang Ho Hospital, New Taipei City, 235, Taiwan

^8^ Department of Medical Laboratory Science and Biotechnology, Yuanpei University of Medical Technology, Hsinchu City 30015, Taiwan;

^9^ Department of Radiation Oncology, Tri-Service General Hospital, National Defense Medical Center, Taipei City, 114, Taiwan.

^#^ Co-First/Equal authorship

* Corresponding Author:

Chun-Shu Lin, MD. Associate Professor Department of Radiation Oncology, Tri-Service General Hospital, National Defense Medical Center, Taipei City, 114, Taiwan

Phone: +886-2-87927192. FAX: 886-2-87927193 E-mail: [chunshulin@gmail.com](mailto:chunshulin@gmail.com)

**Running title: Bruton tyrosine kinase as a therapeutic strategy for OSCC**

**
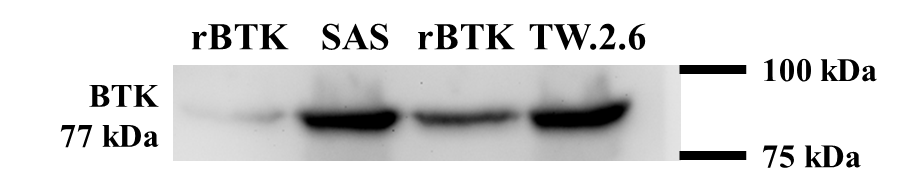
**

**Supplementary Figure S1.** Western blot analysis loading the purified 77kDa rBTK to determine which isoform is overexpressed in OSCC cell lines.

**
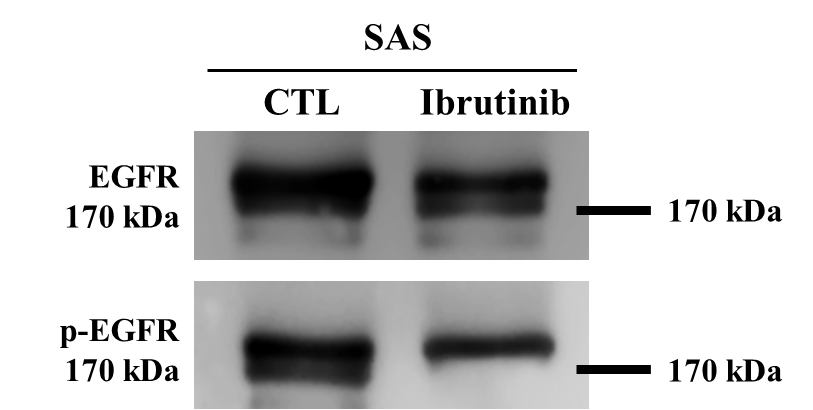
**

**Supplementary Figure S2.** Ibrutinib reduces the expression of EGFR phosphorylation in OSCC cells.
