# Supplementary material for: Inhibition of Bruton’s tyrosine kinase as a therapeutic strategy for chemoresistant oral squamous cell carcinoma and potential suppression of cancer stemness
Source: Oncogenesis. 2021 Feb 27;10(2):20. doi: 10.1038/s41389-021-00308-z (PMC7914253; doi:10.1038/s41389-021-00308-z)
Supplement: Supplementary file 2 — Conflict of Interest Statement [file 41389_2021_308_MOESM2_ESM.docx]

Conflict of Interest Statement (February 3, 2021)

All authors are working for either university or hospitals. We claim that we do not have any actual or potential conflict of interest including any financial, personal or other relationships with other people or organizations within three years of beginning the work submitted that could inappropriately influence our work.

**Inhbition of bruton tyrosine kinase as a therapeutic strategy for chemo-resistant oral squamous cell carcinoma and potential suppressor of cancer stemness**

Disclosed by Shao-Cheng Liu^1#^, Yang-Che Wu^2,3#^, Chih-Ming Huang^4^, Ting-Yi Huang^5,6^, Chin-Sheng Huang ^2,3^, Tung-Nien Hsu^2,3^, Mao-Suan Huang^2,3^, Wei-Hwa Lee^6,7^, Chi-Tai Yeh^6,7,8#^, Chun-Shu Lin^9*^

Authors’ affiliation:

^1^ Department of Otolaryngology-Head and Neck Surgery, Tri-Service General Hospital, National Defense Medical Center, Taipei City, 114, Taiwan;

^2^ School of Dentistry, College of Oral Medicine, Taipei Medical University, Taipei City 110, Taiwan;

^3^ Department of Dentistry, Taipei Medical University - Shuang Ho Hospital, New Taipei City, 235, Taiwan;

^4^ Department of Otolaryngology, Taitung Mackay Memorial Hospital, Taiwan

^5^ Department of Hematology and Oncology, Cancer Center, Taipei Medical University - Shuang Ho Hospital, New Taipei City, 235, Taiwan;

^6^ Department of Medical Research & Education, Taipei Medical University - Shuang Ho Hospital, New Taipei City, 235, Taiwan;

^7^ Department of Pathology, Taipei Medical University-Shuang Ho Hospital, New Taipei City, Taiwan

^8^ Department of Medical Laboratory Science and Biotechnology, Yuanpei University of Medical Technology, Hsinchu City 30015, Taiwan;

^9^ Department of Radiation Oncology, Tri-Service General Hospital, National Defense Medical Center, Taipei City, 114, Taiwan.

^#^ Co-First/Equal authorship

* Corresponding Author:

Chun-Shu Lin, MD. Associate Professor Department of Radiation Oncology, Tri-Service General Hospital, National Defense Medical Center, Taipei City, 114, Taiwan

Phone: +886-2-87927192. FAX: 886-2-87927193 E-mail: [chunshulin@gmail.com](mailto:chunshulin@gmail.com)
