# Supplementary figures and images for: Inhibition of Bruton’s tyrosine kinase as a therapeutic strategy for chemoresistant oral squamous cell carcinoma and potential suppression of cancer stemness
Source: Oncogenesis. 2021 Feb 27;10(2):20. doi: 10.1038/s41389-021-00308-z (PMC7914253; doi:10.1038/s41389-021-00308-z)

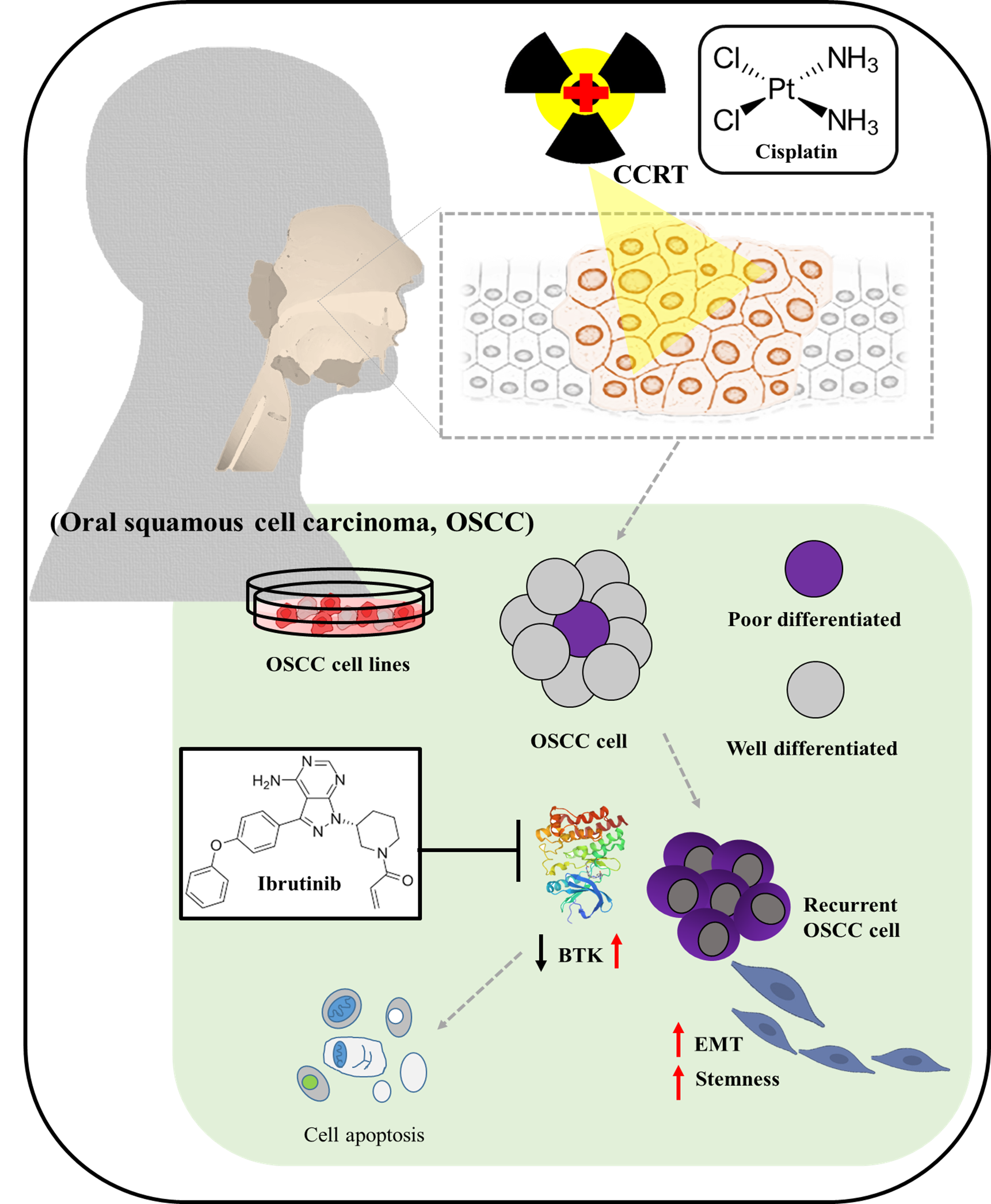


**Pictorial Abstract**

Supplement: Supplementary file 3 — Graphical Abstract [file 41389_2021_308_MOESM3_ESM.docx]
