# Supplementary material for: Inhibition of Bruton’s tyrosine kinase as a therapeutic strategy for chemoresistant oral squamous cell carcinoma and potential suppression of cancer stemness
Source: Oncogenesis. 2021 Feb 27;10(2):20. doi: 10.1038/s41389-021-00308-z (PMC7914253; doi:10.1038/s41389-021-00308-z)
Supplement: Supplementary file 4 — Highlights [file 41389_2021_308_MOESM4_ESM.docx]

**Highlights**

1. BTK was aberrantly expressed in clinical CCRT-resistant OSCC
2. Ibrutinib reduced the ALDH-rich OSCC cells
3. Combination use of ibrutinib and cisplatin had significant tumor suppression effect on ALDH+ OSCC.
